# Problematic social media use, digital health literacy, and psychological distress in Saudi adolescents

**DOI:** 10.3389/fpubh.2026.1926618

**Published:** 2026-10-02

**Authors:** Khaled A. Shahat

**Affiliations:** 1 Department of Women and Child Health, College of Medicine, Taibah University, Madinah, Saudi Arabia; 2 Al-Rayan National College of Medicine, Madinah, Saudi Arabia

**Keywords:** adolescent, digital health literacy, mental health, Saudi Arabia, social media

## Abstract

**Objectives:**

This cross-sectional study examined the prevalence of problematic social media use (PSMU) among Saudi adolescents and its associations with digital health literacy (DHL) and psychological distress (depression, anxiety, and stress).

**Methods:**

A total of 330 Saudi adolescents (170 male, 160 female) aged 13–18 years from intermediate and secondary schools in Madinah, Saudi Arabia, completed validated Arabic versions of the Bergen Social Media Addiction Scale (BSMAS), the Digital Health Literacy Instrument (DHLI), and the Depression, Anxiety, and Stress Scale (DASS-21). Multivariate logistic regression analyses were performed to identify factors associated with PSMU and its mental health correlates.

**Results:**

The prevalence of PSMU was 34.8% (95% CI: 29.7–40.2%), with significantly higher rates among females compared to males (41.9% vs. 28.2%, *p* = 0.009). Low DHL was independently associated with PSMU (adjusted OR = 2.79, 95% CI: 1.70–4.58, *p* < 0.001). Participants with PSMU had significantly higher mean scores on all DASS-21 subscales compared to non-problematic users (depression: 14.1 ± 5.5 vs. 8.6 ± 4.2, *p* < 0.001; anxiety: 12.7 ± 5.0 vs. 7.8 ± 3.9, *p* < 0.001; stress: 15.5 ± 5.7 vs. 10.0 ± 4.6, *p* < 0.001). PSMU was significantly associated with moderate-to-severe depression (aOR = 3.18, 95% CI: 1.88–5.38), anxiety (aOR = 2.90, 95% CI: 1.72–4.89), and stress (aOR = 2.64, 95% CI: 1.56–4.47).

**Conclusion:**

PSMU is highly prevalent among Saudi adolescents in Madinah and is strongly associated with low digital health literacy and adverse mental health outcomes. These cross-sectional findings demonstrate associations between PSMU, low digital health literacy, and adverse mental health outcomes, suggesting the potential value of school-based digital literacy interventions and mental health screening programmes. However, longitudinal studies are needed to establish causal relationships before definitive intervention recommendations can be made.

## Introduction

The proliferation of digital technologies has fundamentally transformed the developmental landscape of adolescence worldwide. In Saudi Arabia, where internet penetration exceeds 99% and social media platforms are extensively used, adolescents represent one of the most digitally engaged demographic groups. A study conducted in 2017 showed that 85% of Saudi adolescents used recreational media excessively (>2 h per day), and more recent data indicate that 86.2% exceed media use recommendations (1). Arab youth are among the most digitally engaged in the world; in Saudi Arabia, adolescents spend between six to ten hours daily online, with gaming, social media, and streaming constituting the primary activities.

While social media offers opportunities for social connection, identity exploration, and information access, accumulating evidence suggests that problematic use patterns are associated with adverse mental health outcomes. Studies from various contexts have linked excessive social media use to increased depression, anxiety, stress, and loneliness among adolescents (2–4). The prevalence of social media disorder among Saudi high school students has been reported to range from 3.3 to 20.7%, depending on the classification approach (2). A national survey of nearly 3,000 high school students across Saudi Arabia found that frequent social media use was associated with higher levels of loneliness and mental distress; nearly a third of students screened positive for loneliness, and over 40% screened positive for depression or anxiety symptoms (2). Internet addiction among Saudi adolescents has been reported at 68% for mild to severe addiction, with adolescents with higher addiction scores perceiving more stress, anxiety, and depression (3). The prevalence of social media addiction among approached Saudis has been estimated at 35.9% (4).

Digital health literacy (DHL) defined as the ability to seek, find, understand, and appraise health information from electronic sources and apply the knowledge gained to addressing or solving a health problem, represents a potentially critical protective factor. In the Saudi context, studies have identified significant variations in digital health literacy across different genders and age groups (5). Individuals with higher DHL are better equipped to critically evaluate online content, recognise misinformation, and make informed decisions about their digital consumption behaviours. However, the relationship between DHL and problematic social media use among adolescents remains underexplored, particularly in the Saudi context.

Previous research in Saudi Arabia has examined various aspects of adolescent health, including depression predictors (6), mental illness prevalence (7), physical inactivity (8), sleep quality (9), cognitive functions (10), and eating disorders (11). Studies have also investigated social media addiction and its correlates in various regions, including Aseer, Jeddah, and nationally (2, 4). However, no published study to date has comprehensively examined the interplay between problematic social media use, digital health literacy, and Psychological Distress among Saudi adolescents in Madinah using a unified framework. This represents a significant gap in the literature, given the rapid digital transformation occurring in the Kingdom and the critical developmental period of adolescence.

Therefore, this study aimed to: (1) estimate the prevalence of problematic social media use among Saudi adolescents in Madinah; (2) examine the association between digital health literacy and problematic social media use; and (3) investigate the relationship between problematic social media use and Psychological Distress outcomes, including depression, anxiety, and stress.

## Materials and methods

### Study design and setting

This was a cross-sectional study conducted between January and March 2026 in Madinah, Saudi Arabia. Participants were recruited from four intermediate and secondary schools (two male, two female) selected through stratified random sampling to ensure representation across different socioeconomic districts of the city.

### Participants

Sample size was calculated using the formula for cross-sectional studies: *n* = (Z^2^ × p × q)/d^2^, where Z = 1.96 (95% confidence level), *p* = expected prevalence of problematic social media use (35% based on previous Saudi studies), q = 1-p, and d = margin of error (5%). This yielded a minimum required sample of 350 participants. To account for an anticipated 10% non-response or incomplete data, we aimed to recruit 385 participants. However, due to time constraints and school scheduling limitations during the data collection period (January–March 2026), the final sample comprised 330 participants (86% of the target). This sample size still provided sufficient power (80%) to detect the primary associations of interest, as the achieved prevalence estimate of 34.8% fell within the expected range.

### Recruitment process and study flow

Four schools (two intermediate, two secondary; one male and one female school per level) were selected through stratified random sampling from the list of all public schools in Madinah city, stratified by socioeconomic district (low, medium, high) based on municipal zoning data. The school principals were contacted and provided with information about the study objectives and procedures. Following school approval, information sheets and consent forms were distributed to students to take home to their parents/guardians. Students who returned signed parental consent forms and provided their own written assent were eligible to participate. Data collection sessions were scheduled during designated school hours in each school over a two-week period. Research assistants distributed the paper-based questionnaires in classrooms, explained the instructions verbally, and remained present throughout to answer questions. Questionnaires took approximately 20–25 min to complete. Completed questionnaires were collected immediately and checked for completeness by research assistants, resulting in no missing data. A total of 385 students were invited to participate; 330 completed the questionnaires (response rate: 85.7%). The primary reasons for non-participation were failure to return signed parental consent forms (*n* = 42) and absenteeism on the day of data collection (*n* = 13).

Inclusion criteria were: (1) Saudi nationality; (2) age 13–18 years; (3) enrolment in intermediate or secondary school; (4) active social media user (at least one account); and (5) willingness to provide informed assent (with parental consent). Exclusion criteria were: (1) diagnosed psychiatric disorders requiring active treatment; (2) cognitive or learning disabilities that would preclude questionnaire completion; and (3) non-Saudi nationality.

### Data collection

Data were collected using self-administered paper-based questionnaires distributed during school hours. Research assistants were present to explain the study objectives and provide assistance with questionnaire completion if needed. Questionnaires were completed anonymously, and participants were assured of confidentiality.

### Instruments

#### Problematic social media use (PSMU)

The Bergen Social Media Addiction Scale (BSMAS) was used, consisting of 6 items rated on a 5-point Likert scale (1 = very rarely to 5 = very often). Consistent with the instrument’s purpose as a screening tool rather than a diagnostic measure, we use the term ‘problematic social media use’ (PSMU) throughout this manuscript to reflect the dimensional nature of the construct and to avoid clinical diagnostic connotations. The BSMAS assesses core features of excessive and dysregulated social media engagement, but a positive screen does not equate to a formal diagnosis of addiction. Items assess core addiction criteria, including salience, mood modification, tolerance, withdrawal, conflict, and relapse. A total score of ≥18 (mean score ≥3) was used to define PSMU, consistent with the original BSMAS validation studies and widely used in the international literature. The Arabic version of the BSMAS has demonstrated good psychometric properties (Cronbach’s α = 0.74–0.87) in an Arabic-speaking university student population (12). However, we acknowledge that the specific ≥18 cutoff has not been formally validated in an Arabic adolescent population. To address this, we conducted sensitivity analyses using alternative cut-offs (≥17 and ≥19), which yielded consistent patterns of association (data not shown). We recommend future population-specific validation studies to confirm the optimal cutoff for Saudi adolescents.

#### Digital health literacy (DHL)

The Digital Health Literacy Instrument (DHLI) was employed, comprising 7 items assessing competencies in: (1) finding health information online; (2) understanding health information; (3) appraising information reliability; (4) determining relevance; (5) navigating websites; (6) interacting with online health content; and (7) adding self-generated content. Items were rated on a 4-point scale (1 = very easy to 4 = very difficult). Lower scores indicate higher DHL.

#### Psychological distress

The Depression, Anxiety, and Stress Scale (DASS-21) was used, consisting of 21 items divided into three subscales of 7 items each. Items are rated on a 4-point scale (0 = did not apply to me at all to 3 = applied to me very much or most of the time). Subscale scores were calculated by summing the item responses for each of the three DASS-21 subscales (depression, anxiety, and stress), each consisting of 7 items with scores ranging from 0 to 21. To apply the standard DASS-42 severity cut-offs (normal, mild, moderate, severe, extremely severe), subscale scores were multiplied by 2 (i.e., depression: 0–9 normal, 10–13 mild, 14–20 moderate, 21–27 severe, ≥28 extremely severe; anxiety: 0–7 normal, 8–9 mild, 10–14 moderate, 15–19 severe, ≥20 extremely severe; stress: 0–14 normal, 15–18 mild, 19–25 moderate, 26–33 severe, ≥34 extremely severe). For the multivariate regression analyses, outcomes were dichotomised as ‘moderate-to-severe’ (moderate, severe, or extremely severe) versus ‘normal-to-mild’ (normal or mild). The Arabic DASS-21 has been validated in Saudi populations and shown good psychometric properties (13).

#### Sociodemographic Variables

Data were collected on age, gender, school grade, parental education, household income, daily social media usage time, and number of social media platforms used.

### Statistical analysis

Data were analysed using IBM SPSS Statistics for Windows, version 26.0 (IBM Corp., Armonk, NY, United States). Descriptive statistics were calculated for all sociodemographic and study variables. Continuous variables (age, daily usage hours, platform count, DHL score, BSMAS score, and DASS-21 subscales) were tested for normality using the Shapiro–Wilk test and visual inspection of Q-Q plots. Normally distributed data were expressed as means ± standard deviations (SD), and non-normally distributed data were reported as medians with interquartile ranges (IQR) where applicable. Categorical variables (gender, education level, parental education, income, and PSMU status) were presented as frequencies and percentages.

For bivariate comparisons between the PSMU and non-PSMU groups, independent-samples t-tests were employed for continuous variables that met the assumptions of normality and homogeneity of variance (Levene’s test). The chi-square (χ^2^) test was used to compare categorical variables between the two groups.

To identify factors independently associated with PSMU (binary outcome: yes/no), we performed multivariate logistic regression analysis. First, a univariate screening was conducted; all variables with a *p*-value < 0.20 in the univariate analysis, along with clinically relevant variables (age and gender), were entered as candidates into the multivariate model. The following independent variables were entered into the multivariate logistic regression model using the ‘Enter’ method: age (continuous), gender (male as reference), education level (intermediate as reference), father’s education (below university as reference), mother’s education (below university as reference), household income (low/medium as reference), daily usage hours (dichotomised at ≥6 h), number of platforms used (dichotomised at ≥5 platforms), and DHL scores were dichotomised at the upper tertile (≥18) to define ‘low digital health literacy’ for the primary analysis. This cut-off was selected empirically based on the distribution of scores in our sample, as there is no established validated cut-off for the DHLI in Arabic adolescent populations. Participants in the upper tertile (≥18) demonstrated the poorest self-reported digital health literacy competencies (i.e., greatest difficulty in finding, understanding, appraising, and applying online health information), allowing meaningful comparison between adolescents with relatively low versus higher DHL. To ensure robustness, we also conducted sensitivity analyses treating DHL as a continuous variable in logistic regression models; these produced consistent findings (data not shown), supporting the validity of the dichotomisation approach.

Similarly, three separate multivariate logistic regression models were constructed to examine the association between PSMU and each mental health outcome: moderate-to-severe depression, moderate-to-severe anxiety, and moderate-to-severe stress (each coded as binary using the standard DASS-21 cut-offs). These models were adjusted for the same set of covariates (age, gender, parental education, income, and DHL score) to isolate the independent effect of PSMU.

Collinearity among independent variables was assessed using variance inflation factors (VIF); a VIF < 5 was considered acceptable, indicating no significant multicollinearity. Model calibration and goodness-of-fit for each logistic regression were evaluated using the Hosmer–Lemeshow test, with a *p*-value > 0.05 indicating adequate fit. The Nagelkerke R^2^ was calculated to estimate the proportion of variance explained by each model. Results from the multivariate analyses were reported as adjusted odds ratios (aOR) with their corresponding 95% confidence intervals (CIs).

All questionnaires were reviewed for completeness by the research assistants at the time of data collection; thus, there were no missing data, and all 330 participants were included in the final analysis without the need for imputation. A two-tailed *p*-value < 0.05 was considered statistically significant for all tests.

### Ethical considerations

The study received ethical approval from the Institutional Review Board of Taibah University, Madinah region (IRB no. 180–26, dated 01-01-2026). Written informed consent was obtained from parents or legal guardians, and written assent was obtained from all adolescent participants. The study was conducted in accordance with the Declaration of Helsinki.

This study is reported in accordance with the Strengthening the Reporting of Observational Studies in Epidemiology (STROBE) guidelines for cross-sectional studies (25).

## Results

### Statistical analysis overview

All analyses were conducted using IBM SPSS Statistics for Windows, version 26.0 (IBM Corp., Armonk, NY, United States). The Shapiro–Wilk test indicated that all continuous variables were approximately normally distributed (*p* > 0.05 for all), with Q-Q plots confirming visual normality; therefore, parametric tests were used for continuous variables. There were no missing data for any variable; all 330 participants were included in the final analysis. Daily usage hours were dichotomised at ≥6 h (median = 5.8 h), platform count at ≥5 platforms (median = 4.0, IQR = 3.0–5.0), and DHL score at ≥18 and DHL score at ≥18 (upper tertile cut-off). On univariate analysis, variables achieving *p* < 0.20 included gender, DHL score, daily usage hours, number of platforms, and mother’s education (data not shown). These were entered into the multivariate model along with age and gender. Multicollinearity assessment revealed VIF values ranging from 1.12 to 2.43, indicating no significant collinearity among the independent variables. The Hosmer–Lemeshow test indicated adequate model fit for all three logistic regression models (*p* > 0.05 for each). Nagelkerke R^2^ values were 0.27 for the depression model, 0.23 for the anxiety model, and 0.21 for the stress model.

### Participant characteristics

A total of 330 adolescents participated in the study, with a mean age of 15.4 ± 1.6 years (range 13–18). The sample comprised 170 males (51.5%) and 160 females (48.5%). The majority were enrolled in secondary school (62.4%, *n* = 206), with 37.6% (*n* = 124) in intermediate school. Parental education levels were: father, university degree or higher (58.2%), mother, university degree or higher (52.1%). Approximately 45.5% (*n* = 150) reported a monthly household income exceeding SAR 15,000.

Regarding social media usage, participants reported a mean daily usage time of 5.8 ± 2.3 h. The most commonly used platforms were Snapchat (92.4%), Instagram (87.6%), TikTok (79.1%), and X/Twitter (65.5%). The mean number of platforms used was 4.2 ± 1.5 (median = 4.0, IQR = 3.0–5.0).

### Prevalence of problematic social media use

The overall prevalence of PSMU was 34.8% (95% CI: 29.7–40.2%). The prevalence was significantly higher among females (41.9%, *n* = 67) compared to males (28.2%, *n* = 48; χ^2^ = 6.82, *p* = 0.009). No significant differences were observed by age group (13–15 years: 32.9% vs. 16–18 years: 36.8%, *p* = 0.46) or school level (intermediate: 33.1% vs. secondary: 35.9%, *p* = 0.61).

### Digital health literacy and its association with PSMU

The mean DHL score was 16.7 ± 4.1 (range 7–28), with lower scores indicating higher literacy. Participants with PSMU had significantly higher DHL scores (indicating lower literacy) compared to non-problematic users (19.3 ± 3.7 vs. 15.3 ± 3.8, *p* < 0.001). After adjusting for age, gender, parental education, and daily usage time, low DHL (defined as score ≥18) remained independently associated with PSMU (aOR = 2.79, 95% CI: 1.70–4.58, *p* < 0.001) (Table 1).

**Table 1:** Factors associated with problematic social media use (multivariate logistic regression).

Variable	Adjusted OR	95% CI	*p*-value
Female gender (ref: male)	1.94	1.17–3.22	0.010
Age (per year)	1.08	0.92–1.27	0.326
Education level (ref: intermediate)			
Secondary school	1.15	0.69–1.92	0.592
Father’s education (ref: below university)			
University or higher	0.81	0.48–1.36	0.424
Mother’s education (ref: below university)			
University or higher	0.67	0.40–1.11	0.128
Household income (ref: low/medium)			
High (>SAR 15,000)	0.78	0.46–1.32	0.354
Daily usage ≥6 h (ref: <6 h)	2.38	1.43–3.96	0.001
Number of platforms ≥5 (ref: <5 platforms)	1.76	1.06–2.93	0.028
Low digital health literacy (DHL ≥18) (ref: DHL <18)	2.79	1.70–4.58	<0.001

All variables were entered simultaneously using the ‘Enter’ method. Ref = reference category. The full model included all variables listed in the Methods section; only statistically significant predictors are shown for clarity.

### Psychological distress outcomes

Participants with PSMU had significantly higher mean scores across all DASS-21 subscales compared to non-problematic users (Table 2).

**Table 2:** Comparison of DASS-21 scores between PSMU and non-PSMU groups.

DASS-21 subscale	PSMU group (*n* = 115)	Non-PSMU group (*n* = 215)	*p*-value
Depression	14.1 ± 5.5	8.6 ± 4.2	<0.001
Anxiety	12.7 ± 5.0	7.8 ± 3.9	<0.001
Stress	15.5 ± 5.7	10.0 ± 4.6	<0.001

The proportion of participants with moderate-to-severe symptoms was significantly higher in the PSMU group across all domains: depression (47.8% vs. 18.1%, *p* < 0.001), anxiety (42.6% vs. 16.3%, *p* < 0.001), and stress (45.2% vs. 19.5%, *p* < 0.001).

After adjusting for potential confounders (age, gender, parental education, household income, and DHL), PSMU remained significantly associated with moderate-to-severe depression (aOR = 3.18, 95% CI: 1.88–5.38), anxiety (aOR = 2.90, 95% CI: 1.72–4.89), and stress (aOR = 2.64, 95% CI: 1.56–4.47) (Table 3).

**Table 3:** Association between PSMU and mental health outcomes (multivariate logistic regression).

Outcome	Adjusted OR	95% CI	*p*-value
Moderate-to-severe depression	3.18	1.88–5.38	<0.001
Moderate-to-severe anxiety	2.90	1.72–4.89	<0.001
Moderate-to-severe stress	2.64	1.56–4.47	<0.001

Adjusted for age, gender, parental education, household income, and DHL.

## Discussion

This study provides novel evidence on the prevalence of problematic social media use among Saudi adolescents in Madinah and its associations with digital health literacy and psychological distress. To our knowledge, this is the first study to simultaneously examine the interrelationships between problematic social media use, digital health literacy, and psychological distress specifically in an adolescent sample from Madinah, Saudi Arabia. While previous studies have examined these factors individually or in other regions of Saudi Arabia (2, 4, 14), the combination of these three constructs within a single adolescent sample from Madinah has not been previously reported, to our knowledge.

### Prevalence and correlates of problematic social media use

The observed prevalence of PSMU (34.8%) is consistent with rates reported in other Saudi studies, where one-third of approached Saudis were found to be social media addicts (35.9%) (4), and substantially higher than the 3.3–20.7% range reported using stricter diagnostic criteria (2). The higher prevalence among females (41.9% vs. 28.2%) aligns with findings from other studies (2, 15) and may reflect gender differences in social media engagement patterns, with females typically using platforms more intensively for social connection and identity expression. It should be noted that the BSMAS is a screening instrument and the 34.8% prevalence represents the proportion of adolescents scoring above the screening threshold, not a clinical diagnosis of social media addiction.

The strong independent association between low DHL and PSMU (aOR = 2.79) represents a particularly important finding. Adolescents with limited ability to critically evaluate online content may be more susceptible to the addictive design features of social media platforms, including infinite scrolling, algorithmic content recommendation, and notification-driven engagement loops. This suggests that DHL may serve as a protective factor against problematic usage, with implications for intervention design. Educational programmes aimed at enhancing adolescents’ capacity to appraise online information critically could potentially reduce vulnerability to problematic social media use (16–18).

### Mental health implications

The observed associations between PSMU and adverse mental health outcomes are consistent with a growing body of international and Saudi literature (14). The threefold increased odds of moderate-to-severe depression among adolescents with PSMU is clinically significant and adds to mounting concerns about the mental health burden associated with excessive digital engagement. Several mechanisms may explain these associations. First, social media use often displaces sleep, physical activity, and face-to-face social interaction—all of which are protective for mental health (8, 9). Second, exposure to idealized representations of others’ lives on social media can foster social comparison and diminish self-esteem. Third, the constant connectivity may impair emotional regulation and increase vulnerability to stress.

The association with anxiety is also noteworthy, potentially reflecting the role of social media in generating fear of missing out (FoMO), social evaluative concerns, and the pressure to maintain an idealized online persona (14, 19). These findings align with recent research documenting the mediating role of FoMO and social isolation in the relationship between social media use and mental health outcomes (20). The finding that over 40% of the overall sample screened positive for depression or anxiety symptoms is consistent with the sobering mental health burden identified in national surveys (2, 21).

### Implications for policy and practice

The findings have several important implications. First, the high prevalence of PSMU and its strong association with mental health problems underscore the need for routine mental health screening in adolescent populations, particularly focusing on digital behaviour patterns. Second, the association between higher DHL and lower odds of PSMU suggests that school-based digital literacy programmes warrant further investigation as a potential prevention approach. However, as this is a cross-sectional study, we cannot infer that improving DHL will directly reduce PSMU; these findings should be interpreted as hypothesis-generating and require confirmation through longitudinal and intervention studies.

The observed association between higher DHL and lower odds of PSMU is consistent with the hypothesis that digital literacy could play a role in preventing problematic social media use. As an implication of this cross-sectional finding, school-based digital literacy programmes warrant consideration and further evaluation. Such programmes, if developed, would aim to develop adolescents’ capacity to critically evaluate online content, recognise manipulative platform design features, and make intentional decisions about their digital consumption. However, we emphasise that these are implications of the observed associations rather than established causal effects; rigorous intervention studies are needed to determine whether improving DHL actually reduces PSMU. Stakeholder perspectives in Saudi Arabia indicate that educational programmes for parents, school programmes, and regulatory apps receive over 75% support as intervention approaches (22). Contextual adaptation of such interventions has been shown to be feasible in the Saudi setting (16).

Third, the gender disparity in PSMU prevalence suggests that prevention and intervention efforts may need to be gender-sensitive, potentially addressing the different patterns and motivations underlying social media use among male and female adolescents. Finally, the findings support calls for digital wellbeing to be recognised as a public health priority in Saudi Arabia (23), with coordinated efforts involving schools, families, healthcare providers, and technology companies. Early evidence from randomised controlled trials targeting digital wellbeing in Saudi adolescents shows promising results (24). The Saudi Ministry of Health’s recommendation of a maximum of 2 h of media use per day for adolescents provides a framework for such efforts.

### Limitations

Several limitations should be acknowledged. First, the cross-sectional design precludes causal inference regarding the direction of associations. While it is plausible that PSMU contributes to mental health problems, reverse causality or bidirectional relationships are also possible. Second, the reliance on self-reported measures may introduce response bias, particularly regarding sensitive topics such as mental health symptoms. Third, the sample was drawn exclusively from Madinah, which may limit generalisability to other regions of Saudi Arabia with different sociodemographic characteristics; however, our results are similar to those from other regions (20). Fourth, we did not assess for comorbid psychiatric conditions or obtain clinical diagnoses, which would have strengthened the clinical relevance of our findings. Fifth, the use of a screening instrument for PSMU rather than a clinical diagnostic assessment means that some participants classified as having PSMU may not meet full diagnostic criteria for social media addiction.

### Strengths

Despite these limitations, the study has notable strengths. The sample size of 330 with near-equal gender representation (170 male, 160 female) provides adequate statistical power for the analyses conducted. The use of validated, culturally adapted instruments enhances the reliability and validity of the measurements. The simultaneous examination of DHL, PSMU, and multiple mental health outcomes within a single framework provides a more comprehensive understanding of these interrelationships than previous studies that have examined these factors in isolation. Furthermore, this is the first study to investigate these relationships specifically in Madinah, contributing to the regional evidence base.

## Conclusion

This study demonstrates that problematic social media use is highly prevalent among Saudi adolescents in Madinah and is significantly associated with low digital health literacy and elevated depression, anxiety, and stress symptoms. These cross-sectional findings highlight associations that support the potential value of evidence-based interventions, but the recommendations for enhancing digital health literacy, promoting healthy social media use, and providing mental health support should be interpreted as implications of the observed associations rather than demonstrated causal effects. Future research employing longitudinal and intervention designs is needed to establish whether improvements in digital health literacy lead to reductions in problematic social media use and associated mental health symptoms.

## Data Availability

The raw data supporting the conclusions of this article will be made available by the authors, without undue reservation.
